# Defect Engineering in Metal‒Organic Frameworks as Futuristic Options for Purification of Pollutants in an Aqueous Environment

**DOI:** 10.3389/fchem.2021.673738

**Published:** 2021-08-16

**Authors:** Yuhua Cao, Xin Mi, Xiang Li, Bo Wang

**Affiliations:** School of Chemistry, China School of Chemistry, Advanced Research Institute of Multidisciplinary Science, Beijing Institute of Technology, Beijing, China

**Keywords:** defect, water, environment, MOFs, removal

## Abstract

Clean water scarcity is becoming an increasingly important worldwide issue. The water treatment industry is demanding the development of novel effective materials. Defect engineering in nanoparticles is among the most revolutionary of technologies. Because of their high surface area, structural diversity, and tailorable ability, Metal‒Organic Frameworks (MOFs) can be used for a variety of purposes including separation, storage, sensing, drug delivery, and many other issues. The application in wastewater treatment associated with water stable MOF‒based materials has been an emerging research topic in recent decades. Defect engineering is a sophisticated technique used to manufacture defects and to change the geometric framework of target compounds. Since MOFs have a series of designable structures and active sites, tailoring properties in MOFs by defect engineering is a novel concept. Defect engineering can excavate hidden active sites in MOFs, which can lead to better performance in many fields. Therefore, this technology will open new opportunities in water purification processes. However, there has been little effort to comprehensively discuss this topic. In this review, we provide an overview of the development of defect engineered MOFs for water purification processes. Furthermore, we discuss the potential applications of defect engineered materials.

## Introduction

The water contaminant elimination process has aroused great attention in recent years (Ali et al., 2012; Bu et al., 2013; Yang et al., 2017). Because of this, the water treatment industry, which is expected to be worth more than $38 billion by 2025, is looking for new solutions. Here, nanotechnology will open new ground and transform how water is purified (Al-Khateeb et al., 2014; Zhang et al., 2016; Zhao et al., 2016; Oyetade et al., 2018). In order to improve the performance of existing MOFs materials (e.g. UiO‒66, MIL‒100, MIL‒101, MOF‒74, and ZIF‒8), we must think about the question of whether a given framework is performing at its fullest capacity. One of the problems is that many unexploited volumes within the MOFs are inaccessible for some guest compounds (Tsao et al., 2009; Cliffe et al., 2014; Fang et al., 2014). In order to solve this problem, researchers should aim to open the active sites inside the MOFs. One possible approach to achieve this purpose is defect engineering (Maes et al., 2011; Vermoortele et al., 2012a). Defect engineering is a sophisticated technique to manufacture defects and to change the chemical environment of target compounds (Kitagawa et al., 2004; Bonelli et al., 2007; Zhou and Kitagawa, 2014). If the defect structure can be created in a controlled manner and precisely characterized, it can be applied to improve the performance of adsorption and catalysis processes. As a result, this modification can improve the water purification performance. MOFs are a class of compounds consisting of metal ions or clusters coordinated to organic ligands (Rowsell and Yaghi, 2004; Férey, 2008; Furukawa et al., 2013). These organic‒inorganic hybrid crystalline porous materials consist of a regular array of positively charged metal ions surrounded by organic linkers (Zhou et al., 2012; Gangu et al., 2016). Nowadays, the applications of MOFs can be found in several areas including gas separation (Car et al., 2006; Perez et al., 2009; Li et al., 2012; Syrtsova et al., 2012; Wang and Lai, 2013; Liu et al., 2019), energy storage (Lee et al., 2012; Bon, 2017; Wang et al., 2017; Xu et al., 2017), sensing (Zhu et al., 2013; Kumar et al., 2015; Hu et al., 2020; Li et al., 2020; Ma et al., 2020; Chakraborty et al., 2021), and many others (Hasan et al., 2012; Seo et al., 2016; Barpaga et al., 2019; Gwon et al., 2020). There has been an increasing amount of research focusing on the water purification process (Ragab et al., 2016; He et al., 2017; Kadhom and Deng, 2018; Kumar et al., 2018; Wang et al. 2020). Several water pollutants (e.g., heavy metal ions (Fu and Wang, 2011; Al-Rashdi et al., 2013; Abdulla et al., 2019), organic pesticides (Tamara et al., 2005; Michael et al., 2006), toxic chemicals (Angela et al., 2003; Valérie et al., 2004), pharmaceutical and personal care products (Hasanet al., 2012; Azhar et al., 2016), endocrine disrupting chemicals, and per‒and polyfluoroalkyl substances (Liu et al., 2015; Chen et al., 2016)) can be eliminated successfully by MOFs or related materials. However, there is a lot of work still to be done to get a full understanding of the reaction mechanisms. Improving the performance of MOFs based on current water treatment technology is in high demand. In this review, we summarized the recent advances in the applications of defect engineering in water treatment processes. We discussed the fundamental knowledge of defect engineering and existing characterization strategies and provided a comprehensive explanation of the current applications in the water treatment process. Defect engineering can excavate the hidden active sites in MOFs. We believe this review will give more guidance to MOFs’ applications in water purification processes in the future.

**GRAPHICAL ABSTRACT:**
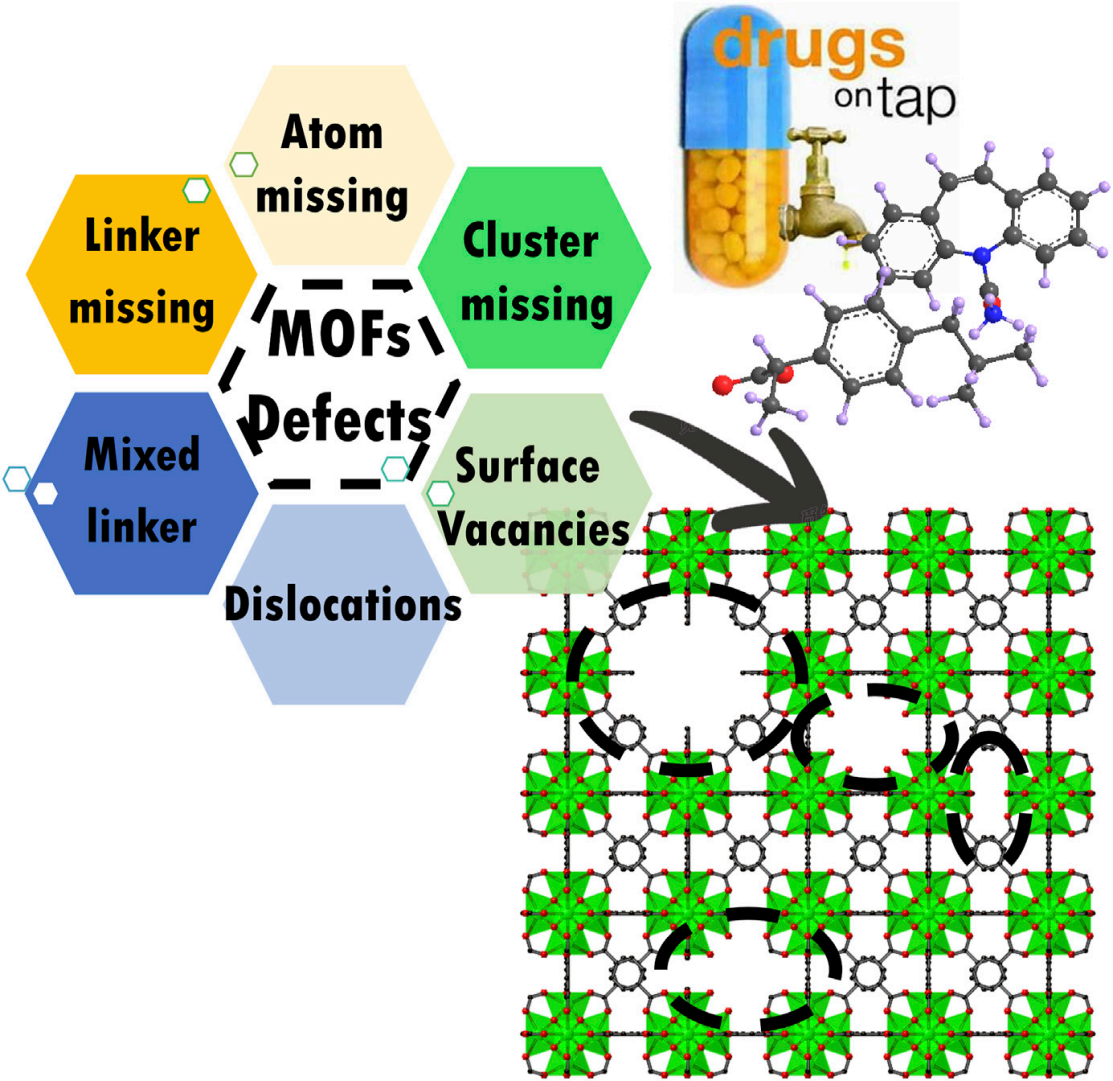


## Definition of Defect Structure

According to recent publications, many types of structural disorders and heterogeneities that disorder the periodic arrangement of atoms in an original structure can be called defect structures. MOFs are a kind of porous polymer which are known to contain defects within their crystalline structure (Shoaee et al., 2008; Shöâeè et al., 2008; Ameloot et al., 2013). This crystalline material has intramolecular pores formed by self‒assembly of metal ions and organic ligands. MOFs have a large specific surface area, adjustable pore size, and are easy to modify (Evans et al., 2014; Lu et al., 2014). These properties have a broad applications prospect in catalysis (Jiao et al., 2018; Li et al., 2018; Xiao and Jiang, 2019), separation (Perez et al., 2009; Li et al., 2012; Wang and Lai, 2013) and purification, gas adsorption, energy sources storage, and many other fields. Combining various metal nodes and organic linkers can produce nearly 70,000 experimentally synthesized MOFs and more than 100,000 hypothetical MOFs. These materials are unique in their chemical composition and topology. A porous crystal skeleton is a highly ordered solid in which the size and shape of all the holes remain uniform, i.e., lattice parameters, atomic symmetries, and coordinates. Uniform pore sizes and holes can be useful in various applications including adsorption, catalysis, and sensing.

However, as with other crystals in nature, an ideal structure with an infinite periodic repetition or ordering of the same atoms in a structure, does not exist. Interestingly, this defect structure can be useful. The heterogeneous crystalline structure also conveys outstanding advantages with long‒range order according to recent research results. For example, changing the building blocks of MOFs within the lattice and guest molecular inside the pores can produce a heterogeneous structure with long‒range order. Introducing different organic linkers, homologous linkers with various functional groups, or different metal‒containing secondary building blocks with the same topological structure are important strategies to improve the properties of materials (Lin et al., 2018; Yuan et al., 2020). During the above processes, defects could be produced with a specific heterogeneous structure, indicating the nonperiodic removal of some structural elements.

## Classification of Defect Structure in Metal‒Organic Frameworks

Here we summarized several methods used to classify the defect structure in MOFs according to recent works.

### Classification 1

**FIGURE 1:**
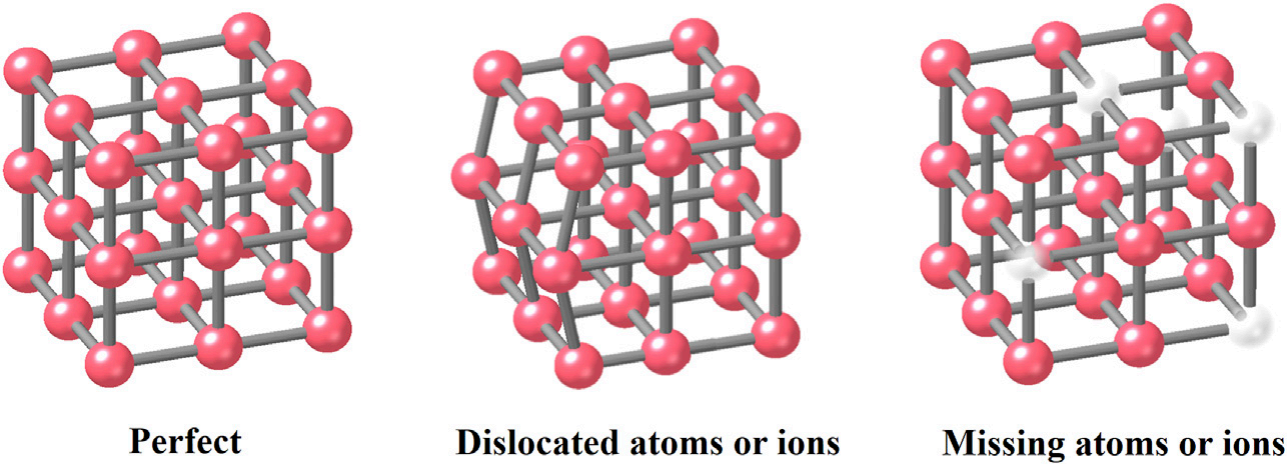
Defect structure classification 1 with missing or dislocated atoms or ions.

Firstly, in recent publications, Roland A. Fischer and his co‒authors defined defects in MOFs as sites that locally break the regular periodic arrangement of atoms or ions of the static crystalline parent framework because of missing or dislocating atoms or ions (Fang et al., 2015) (Figure 1). According to the conclusion in their research groups, there are four major kinds of defects in MOFs: 1) point defects (e.g., vacancies), 2) line defects (e.g., dislocations), 3) planar defects (e.g., boundary or stacking disorders), and 4) micro‒ and mesoscale volume defects (e.g., inclusion and voids) (Barin et al., 2014). Moreover, macroscale volume defects also include the macropores, cracks, and foreign inclusions caused by the synthesis process.

### Classification 2

**FIGURE 2:**
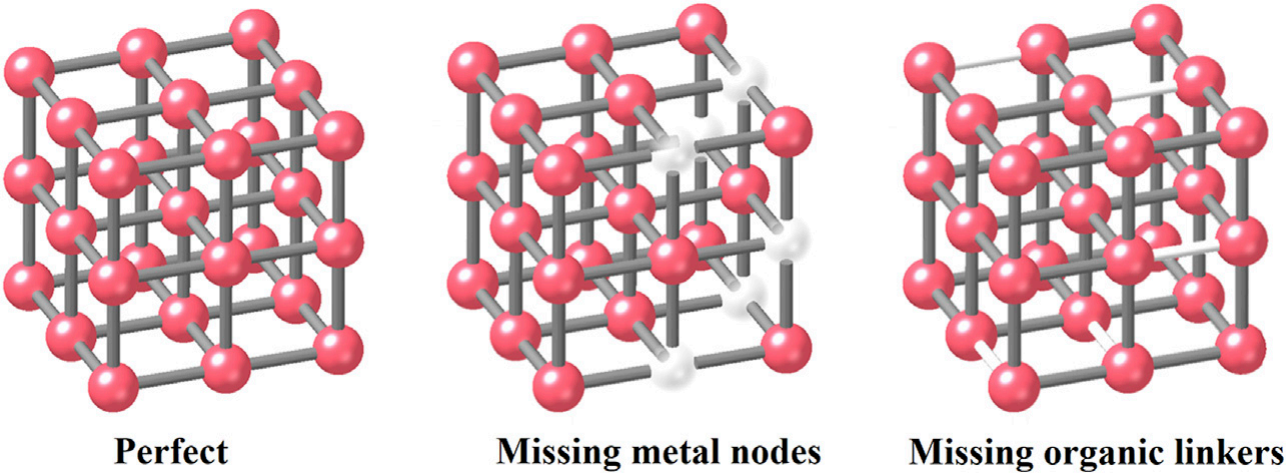
Defect structure classification 2 with missing metal nodes or organic linkers.

Another classification method is based on locations. In this case, defects can be designated as external/surface defects (Vermoortele et al., 2012a; Vermoorteleet al., 2012b) or internal defects (Bordiga et al., 2007; Valvekens et al., 2014). According to published literature, some central metal nodes or organic linkers in MOFs could be missing and locally breaking the framework regularity during the specific synthesized procedures (Kozachuket al., 2013; Fuet al., 2019) (Figure 2). In this case, the linker missing process will form the linker vacancies that may further cause the appearance of modified coordinatively unsaturated metal sites (known as the CUSs). In this way, this defect can also be classified into the point defects 1) in classification 1 discussed above. CUSs are one of the major reaction sites in both adsorption and catalysis technology in water treatment processes. We will give more examples in the following sections.

### Classification 3

**FIGURE 3:**
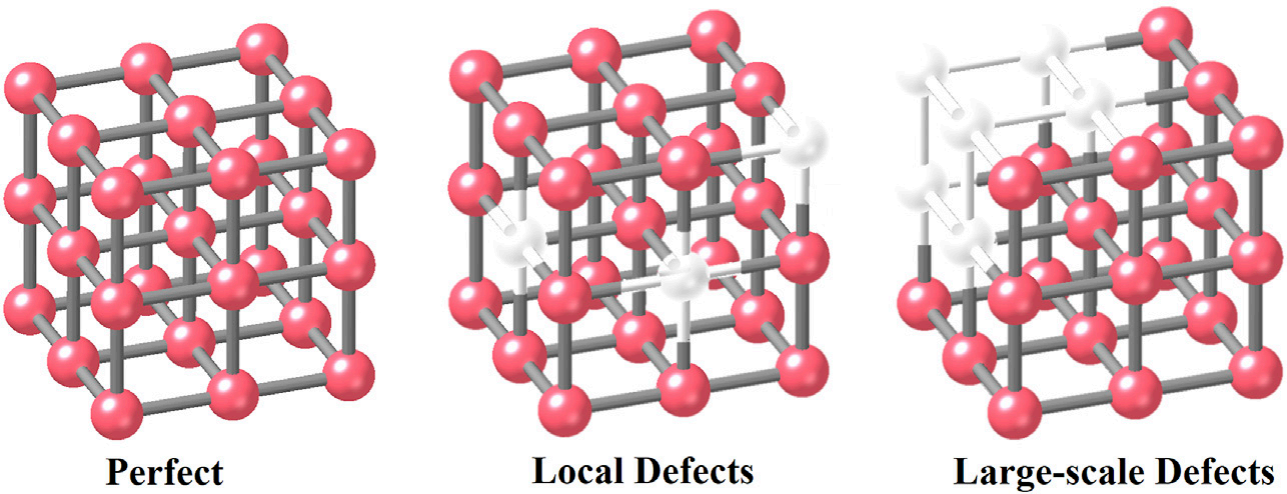
Defect structure classification 3 with defect concentrations.

The third classification method is based on the distributions of defective size (Figure 3). For example, the defects in the framework can be divided into two groups: 1) local defects which point or isolated ones 2) large scale (Cai and Jiang, 2017) with mesoporous. The formation depends on the reaction conditions of specific procedures. High concentrations of defect structures can result in the formation of large‒scale defects through cluster missing (Shearer et al., 2016; Fu et al., 2019). Such large‒scale defects are usually of high dimensions, resulting in the generation of mesoporous structures. For a water treatment process, these linker/cluster vacancies can facilitate the following purification processes:

(1) might greatly enhance mass transfer for water pollutants’ adsorption and catalysis,
(2) might reduce the MOF’s rigidity and increase stability,
(3) might offer CUSs that can facilitate the catalytic reaction selectively.

Here we list some examples of this important type of missing linkers and clusters of defect formation process. Omar K. Farha and his coauthors applied a mixed linker‒fragment approach using parent frameworks NU‒125 with rht topology and HKUST‒1 (Cliffe et al., 2014). Two kinds of cheap fragment acid (part of the structure from original linkers) have been utilized to obtain the mixed linker/copper cluster MOFs, leading to better gas separation performance. In addition, other studies found that CUSs in Cu ions (HKUST‒1) can enhance the gas adsorption process (Bordiga et al., 2007). Other MOFs, including Hf/Zr‒based MOFs with a mesoporous structure caused by missing linkers and missing metal clusters, can change the material’s properties accordingly (Klet et al., 2016). Moreover, defect engineering can be applied in Materials of Institute Lavoisier (MIL) MOFs. For example, MIL‒140 (less than 10% Zr atoms) with CUSs has been synthesized in previous studies (Guillerm et al., 2012). Adding additional Fe^3+^ in MIL‒100 (Fe) can improve the catalytic activity by producing defect structures (Vermoortele et al., 2012b). MOF‒505 (0.57% metal deletions) has also been reported to be synthesized (Shen et al., 2012).

## Formation Strategies and Characterizations

### Formation Strategies

**FIGURE 4:**
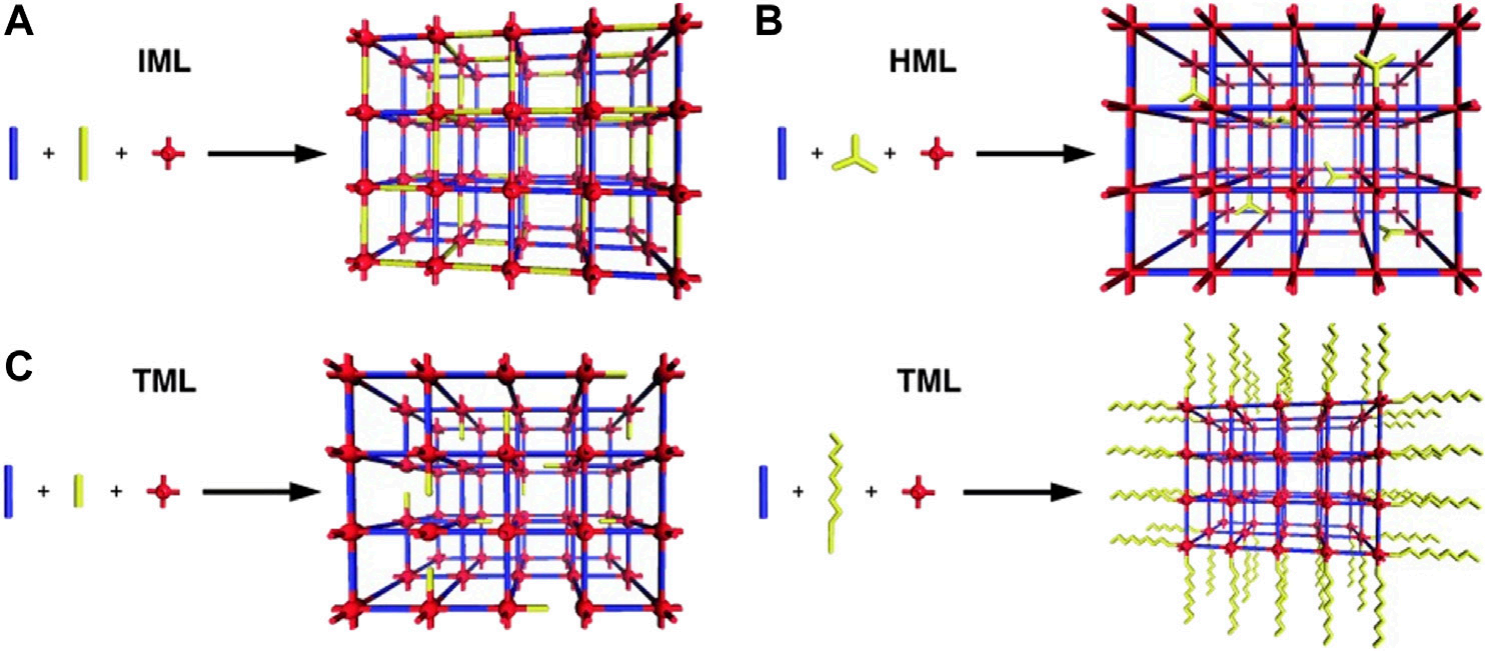
Illustrations of the **(A)** isostructural mixed linker (IML), **(B)** heterostructural mixed linker (HML), and **(C)** two outcomes of truncated mixed linker (TML) approaches to framework functionalization (Bunck and Dichtel, 2013).

Formation strategies for defective MOFs can be divided into two categories. The first one is the direct synthesis method (DSM) (Shearer et al., 2016; Cai and Jiang, 2017), which can be realized by the following processes: 1) by directly mixing two or more organic linkers, such MOFs materials can combine the properties of multiple ligands (Yuan et al., 2020) and the topological structure remains keep unchanged. 2) By adjusting the proportion of precursors and modulators, metal connection points and ligands might be lost. The second one is the post‒synthetic modification (PSM), which can be achieved through the following processes: 1) strategy of acid/base post‒treatment (Vermoortele at al., 2012b). 2) Solvent exchange and subsequent material activation (Tu et al., 2014). The first strategy discussed above can be divided into three parts: isostructural mixed linker (IML), heterostructural mixed linker (HML), and truncated mixed linker (TML) (Bunck and Dichtel, 2013). The simplest and most common method of IML for incorporating multiple monomers into materials is to crystallize them using two or more organic ligands with similar size. The HML method mixes different coordination geometrical shapes. The TML method uses multifunctional monomers to conglutinate with monomers with fewer reactive groups. See Figure 4.

**FIGURE 5:**
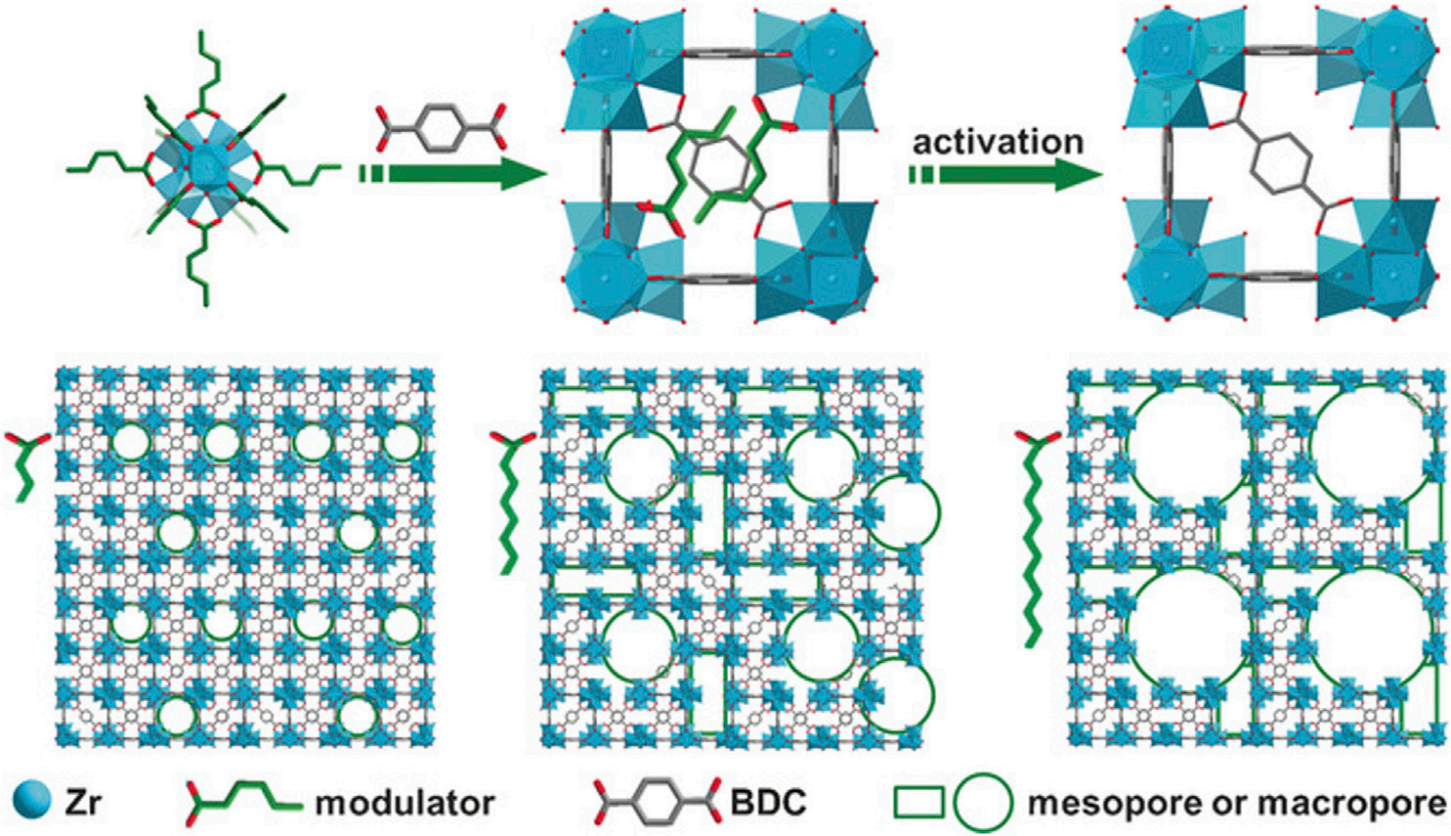
Schematic illustration of the synthesis of HP‒MOFs (UiO‒66) (Cai and Jiang, 2017).

Here we give an example of DSM (Katz et al., 2013). In this case, monocarboxylic acid was added into the reaction system in order to compete with BDC linkers for the coordinated sites on Zr‒O cluster. The pK_a_ of BDC is lower than that of the monocarboxylic acid, making it easier to coordinate with the Zr‒O cluster. This insufficient connection can cause defects (Cai and Jiang, 2017). See Figure 5. Schaate et al. reported that defective UiO‒66 could be synthesized by changing the temperature and modulator (Schaate et al., 2011). Defective MIL‒100 (Fe) can be synthesized by TFA or HClO_4_ treatment (Vermoortele et al., 2012b). Cubitillas et al. reported that defect structure can be made in MOF‒5 under high temperatures (Gadipelli and Guo, 2014). Table 1 lists the representative synthesis methods of defective MOFs.

**TABLE 1:** Representative synthesis methods of defective MOFs.

Materials	Typical strategies	Detailed method	Ref.
MOF‒5	DSM	Mixing ligands	Bunck and Dichtel (2013)
MOF‒5	DSM	Modifying ligands	Deng et al. (2010)
UiO‒66	DSM	Changing temperature	Matthew et al. (2017)
UiO‒66	DSM	Adding regulators	Schaate et al. (2011)
MIL‒100(Fe)	PSM	Acid treatment	Vermoortele et al. (2012b)
UiO‒66	DSM	Heat treatment	Shearer et al. (2014)
MOF‒5	PSM	Heat treatment	Gadipelli and Guo (2014)

### Characterizations

Common methods to characterize the surface properties are as follows: high‒resolution neutron powder diffraction (HRNPD) (Wu e al., 2013), thermogravimetric analysis (TGA), nuclear magnetic resonance (NMR) spectroscopy, atomic force microscopy (AFM), confocal fluorescence microscopy (CFM), scanning electron microscopy (SEM), powder X‒ray diffraction (PXRD), and a combination of different techniques. We will introduce each method in the following section. In the TGA process, the mass loss can be analyzed based on TGA. Increasing the temperature can remove the solvent and hydroxyl from the frameworks. Then the organic ligands are removed with the increasing temperature. Based on this process, the weight loss of the organic ligands can be calculated. NMR spectroscopy is the most powerful tool for organic and organometallic compound determination. In this case we can apply this method to determine the elements of each modulator in the synthesized process. It also can be employed for detecting signs of additional functional groups from organic solvent. For example, Shearer et al. characterized defective UiO‒66 and pristine UiO‒66 with TGA and NMR (Shearer et al., 2016). Moreover, AFM can be applied to study the defect structure and to observe the process of MOF growth. This method can be useful for discovering many features of the material surface as well as the structural defects. For example, Shoaee et al. used high‒resolution AFM to reveal the crystal growth characteristics and defect types of MOFs (HKUST‒1) (Shoaee et al., 2008). Also, CFM, acid‒base titration and SEM should be useful to realize 3D visualization of defects. Christian found that it would be necessary to visualize the defect formation in order to have a better understanding of the performance of MOFs (Christian, 2013). Vermoortele et al. applied CFM to characterize the defects in HKUST‒1 and MOF‒5 (Vermoortele et al., 2012b). In addition, acid‒base titration and SEM are also two important ways to characterize defects (Matthew et al., 2017).

## Applications in Adsorption and Catalysis in Water

Compared with other conventional materials, MOFs with defect structures show several advantages in catalysis and adsorption processes: 1) the high surface areas can reduce the mass transfer by improving the contact opportunities between pollutants and MOFs. 2) Multiple‒functional catalytic sites benefit the catalytic selectivity. The porous structure (mesoporous or macroporous) can be changed by introducing vacancy defects, which can facilitate the adsorption and catalysis process compared to perfect crystal ones. According to recent publications, much of the research focuses on the purification technologies for metal ions, organic pollutants, and emerging contaminants by the water stable Zr‒metal‒organic framework (UiO‒66) of various defects.

### Adsorption

#### Metal Ions

**FIGURE 6:**
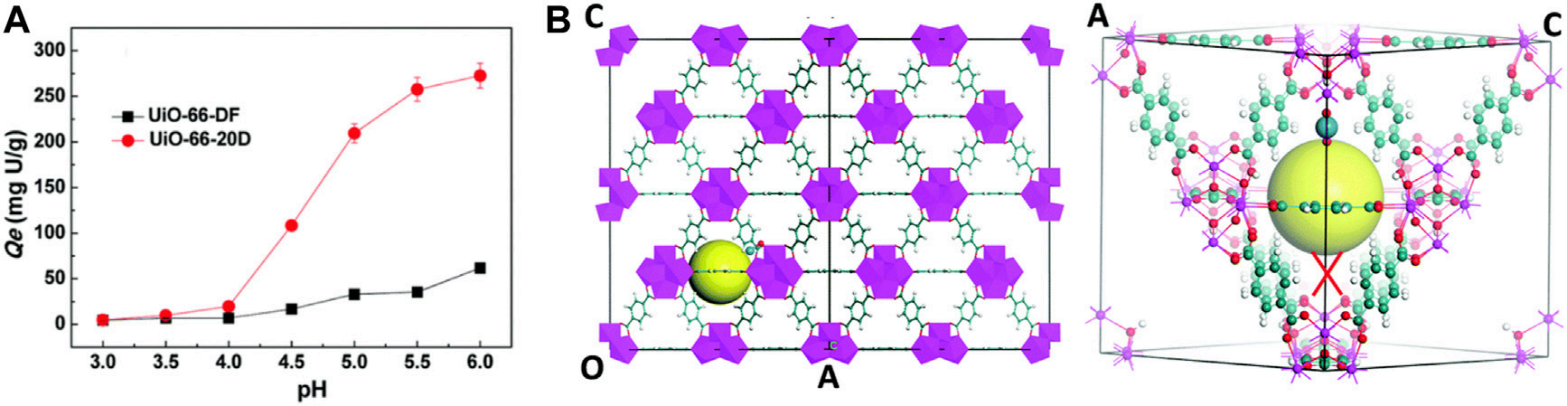
**(A)** Metal ions adsorption on defect free UiO‒66 (UiO‒66‒DF) and defective UiO‒66 (UiO‒66‒20D) and **(B)** proposed mechanisms between uranyl ions and UiO‒66 structure (Yuan et al., 2018).

Recent studies show that defective UiO‒66 can be applied in metal ions separations in the field of nuclear energy. There are increasing active sites (Zr‒OH) and expanding porous structures for binding U (VI) within the framework (Figure 6A). This missing linker defect structure increased the metal ions sorption significantly, as Yuan et al. reported (Yuan et al., 2018). However, all these findings substantiated the applicability of this material for metal ions captured from acidic media. Thus, the defect engineering may provide a new strategy for adsorbing radionuclides. In Figure 6B, the authors first simulated free energy of the equilibrium sorption state of the hydrated uranyl ions. After comparing the two proposed mechanisms based on the same reaction coordinate, the defect structures with one edge of the window broken by removing an aromatic linker showed a lower energy barrier compared to the perfected structures. Therefore, the oxygen atoms at the left‒hand side that are bonding to Zr atoms ultimately form hydroxyl groups. Based on the above discussions, there should be two main mechanisms: 1) the missing‒linker defects in the frameworks expand the narrow pore size by combining neighboring pores. 2) The presence of the defect necessitated the hydroxo or aquo groups to complete the Zr‒coordination sphere, and the linker induced hydroxo groups (Zr‒OH) are very reactive for vapor‒phase metalation. However, there is not enough evidence provided to prove the Zr‒OH groups are the main binding sites for sorption applications. Zr‒OH groups, similar to the Si‒OH groups, have been found to be effective for binding metal ions in the previous report. Therefore, the hydroxo or aquo groups within the defect structure should be one of the mechanisms for metal ions adsorption.

Another piece of research conducted by Yin et al. also confirmed that the defect structure in the framework increased the uranium adsorption kinetics significantly (Yin et al., 2019). They introduced dodecanoic acid in the synthesis and produced hierarchical porous UiO‒66 (HP‒UiO‒66). HP‒UiO‒66 exhibited an ultrarapid adsorption capacity for uranium and reached adsorption equilibrium at 2 min. XPS patterns indicated that two types of Zr‒O bonds (one is μ_3_O, the bridge between Zr‒O‒Zr in zirconium centers and Zr‒H_2_BDC joints) play an important role in uranium adsorption.

**FIGURE 7:**
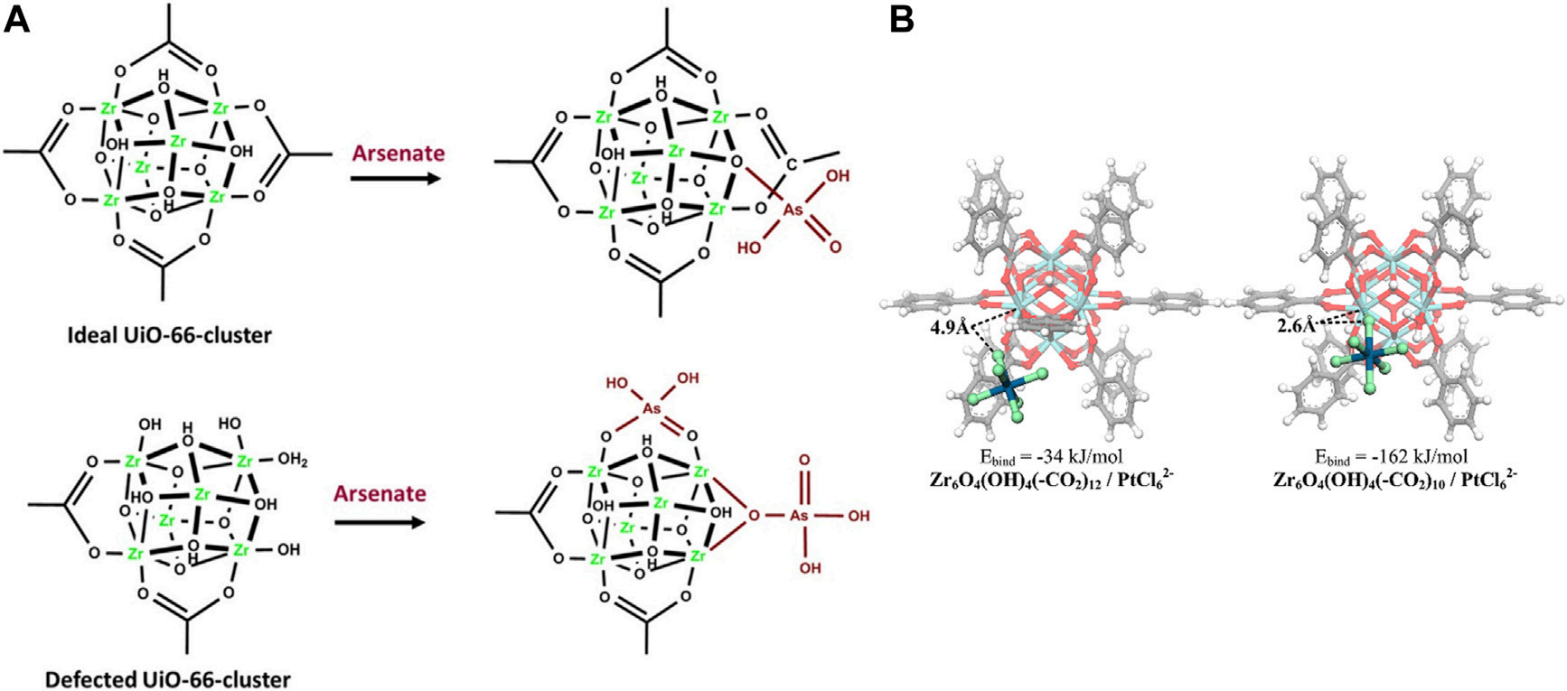
Possible mode of adsorption of **(A)** arsenate on the ideal zirconium and the defected ones (Assaad et al., 2020) and **(B)** Pt (IV) sorption (Lin et al., 2019).

Studies also show that the defective UiO‒66 is effective for other metal ions removal and recovery, such as arsenic and Pt (IV), with acetic acid and trifluoroacetic acid addition as the modulator. The arsenate adsorption capacity increased to 200 mg/g at neutral pH which is the highest value among all the studied samples so far (Assaad et al., 2020). This performance can be contributed to the Lewis acid sites formed in the MOF clusters as a result of missing linker defects. It has been demonstrated that the first adsorption process by a defected free UiO‒66 is the formation of four Zr−OH groups in a unit Zr_6_ cluster. However, in a highly defected UiO‒66 structure, the preferred pathway for arsenate uptake is most likely via adsorption on the missing linker sites on the Zr_6_ nodes via a single or double coordination mode (Figure 7A). Lin et al. synthesized the defective UiO‒66 by controlling the ratio of HCl and ligands in the synthesis, which was used to recover Pt (IV) in strongly acidic solutions (Linet al., 2019). The defective UiO‒66 shows efficient adsorption of Pt (IV). The possible interaction between Pt (IV) and UiO‒66 materials may be the electrostatic attraction or ion exchange between PtCl_6_^2‒^ and the incompletely coordinated Zr sites with terminal ‒OH groups (Figure 7B).

#### Organic Pollutants

Several organic contaminants have been selected as model compounds to study the efficiency of defected‒based materials, such as Rhodamine B (RhB), chemical warfare agents (CWA), and coomassie brilliant blue R250. Fan et al. synthesized a defective ZnIr‒MOF by introducing another ligand, Ir‒BH_3,_ into the matrix (the original ligand is Ir‒AH_3_) (Fan et al., 2017). The defective ZnIr‒MOF has a variety of pore size distributions from micropores to mesopores compared to the uniform micropore distribution in the pristine materials. This material can increase the performance of n‒butanol. Harvey et al. calculated the adsorption interaction between organophosphorus compounds (CWA) and UiO‒66 by density functional theory. Compared with the ideal site with complete coordination, the binding energy at the defect site with unsaturated coordination was increased by nearly two times. Favorable binding is also demonstrated at two additional adsorption sites, Zr‒OH (created sites by a missing linker) and μ_3_‒OH, which likely play a role in the initial adsorption process (Harvey et al., 2018).

**FIGURE 8:**
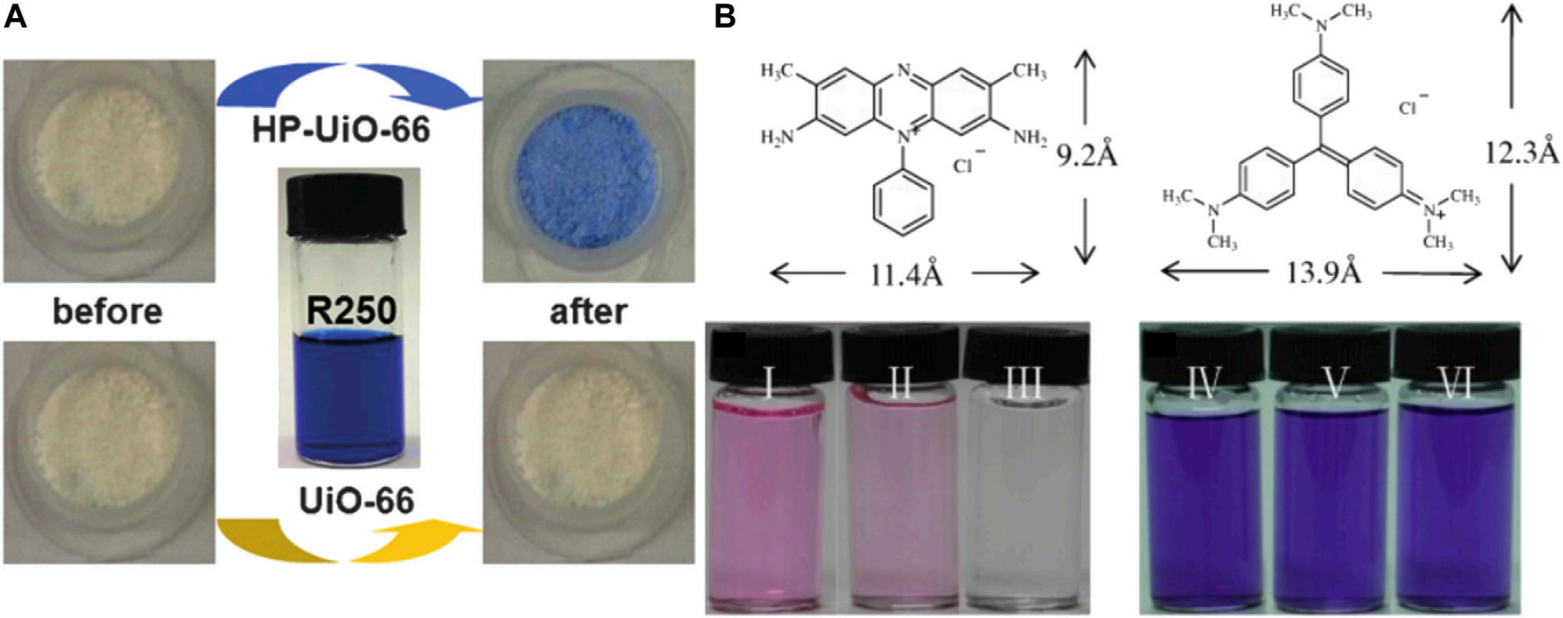
**(A)** Color of the powder sample of UiO‒66 and HP‒UiO‒66 before and after dye adsorption (Cai and Jiang, 2017) and **(B)** photographs of ST and CV before and after adsorption process by defect free UiO‒66 (UiO‒66‒ND) and defective UiO‒66 (UiO‒66‒15) (Wang et al., 2016).

Molecular size and geometry configuration are also important in the process of adsorption by defective materials (Cai and Jiang, 2017). As shown in Figures 8A,B, defective UiO‒66 can be synthesized by adding monocarboxylic acid as a modulator. As shown in Figure 8B, Wang et al. used benzoic acid as a regulator to synthesize defective UiO‒66 (2.7 × 1.7 × 0.9 nm). They used two cationic dyes with different molecular sizes (Safranine T, ST, 9.2 × 11.4 Å in size; Crystal Violet, CV, 12.3 × 13.9 Å in size) as model molecules to explore the effect of defects on the adsorption process. After soaking UiO‒66‒15 in the dye solution, the color of ST solution became obviously colorless, however, the color of CV changed slightly for UiO‒66‒ND (Wanget al., 2016).

In addition, introducing alkaline N‒compounds dopamine, pyrrole, and 2‒methylimidazole can construct defective UiO‒66, as reported by Hu et al. N‒doped UiO‒66 possessed improved alkali‒resistance and higher alkaline surface compared to pristine UiO‒66, and pyrrole‒doped UiO‒66 showed two times enhanced adsorption capacity for RhB (384.1 mg/g) and 223 times higher selectivity for equimolar RhB/ST than that of parent UiO‒66. Alkaline groups on the surface, meaning uniform larger defect pores can enhance the selectivity adsorption performance of cationic dyes (Huet al., 2019).

#### Emerging Contaminants

**FIGURE 9:**
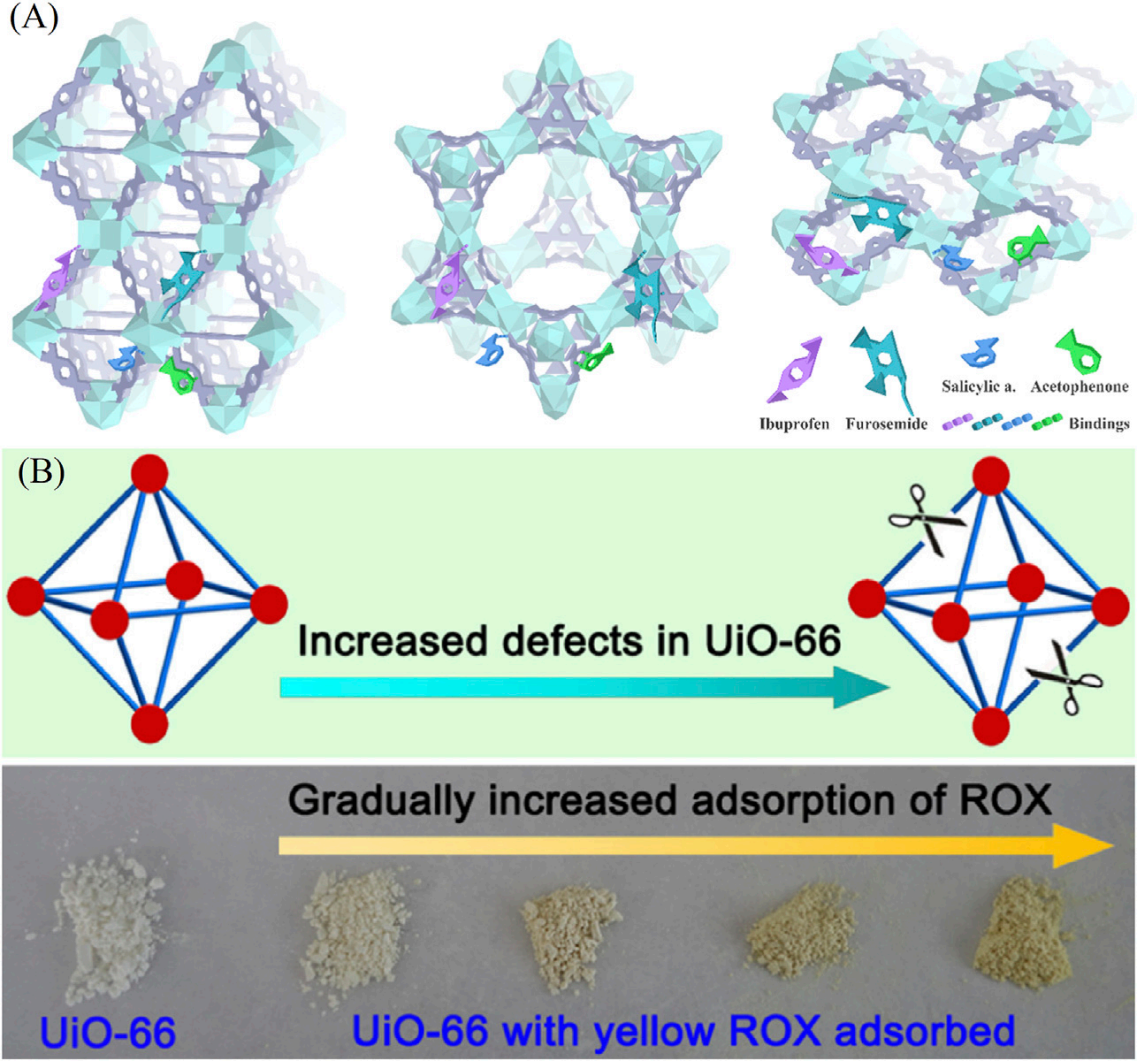
**(A)** Schematic diagram of interactions of ibuprofen, furosemide, salicylic acid, and acetophenone with Zr‒MOFs (Lin et al., 2018) and **(B)** defect tunable UiO‒66 MOFs for ROX removal (Li et al., 2016).

The incompletely coordinated Zr atom in the framework showed a very fast metal adsorption rate according to the published work. The incomplete‒coordinated cationic Zr in the cluster has high affinity for the anionic pharmaceuticals (chemical adsorption), such as ibuprofen, ketoprofen, naproxen, indomethacin, furosemide, and salicylic acid. The terminal ‒OH groups in the incomplete‒coordinated Zr tend to form a positive charged Zr‒OH_2_^+^, and the carboxyl groups in pharmaceuticals were ionized which can lead to a strong electrostatic attraction on the cationic Zr sites (Figure 9A). Porous structures can also influence the number of uptakes for pharmaceuticals, for example, MOF‒802 shows a low uptake rate because of its narrow pores. Different from metal ions, the emerging contaminants have complicated chemical structures including negative charged ‒COO^‒^, tertiary amine groups, and benzene rings. DFT calculations reveal that larger quantities of functional groups (benzene rings) facilitate the adsorption rate by π‒π interaction. π‒π stackings were formed by a strong electronic interaction between good electron donors (π‒electron clouds) and electron acceptors (σ‒framework) (Lin et al., 2018).

Perfluorooctanesulfonate (PFOS) is a persistent organic pollutant that is also toxic. UiO‒66 of several defect concentrations were synthesized. Under the mechanism of electrostatic interaction between PFOS negative head groups and CUSs Zr sites, MOFs had a maximum Langmuir sorption capacity of 1.24 mmol/g, which is two times higher than conventional materials including powdered activated carbons and commercial ion‒exchange resins. Therefore, the enhanced PFOS adsorption indicated that defective UiO‒66 had advantages for contaminated water treatment (Clark et al., 2019). By introducing benzoic acid, Li et al. obtained defective UiO‒66 with missing‒linkers. The defective UiO‒66 can remove a typical organic arsenic compound of roxarsone (ROX) from aqueous solution up to 730 mg/g, which is much higher than those of many reported adsorbents (Figure 9B). The presence of the defects not only resulted in a dramatically enhanced porosity, but also induced the creation of Zr‒OH groups which served as the main active adsorption sites for efficient ROX removal. Another similar work was reported by Xu et al. In addition to the regulation defect, they also changed the ligand and synthesized the defective UiO‒66‒NH_2_ to removal p‒arsanilic acid (p‒ASA) and roxarsone (ROX) (Li et al., 2016). Defect structure and amino groups play an important role in hydrogen bonds formation, which can in turn facilitate the adsorption process.

### Catalysis

#### Photocatalysis

**FIGURE 10:**
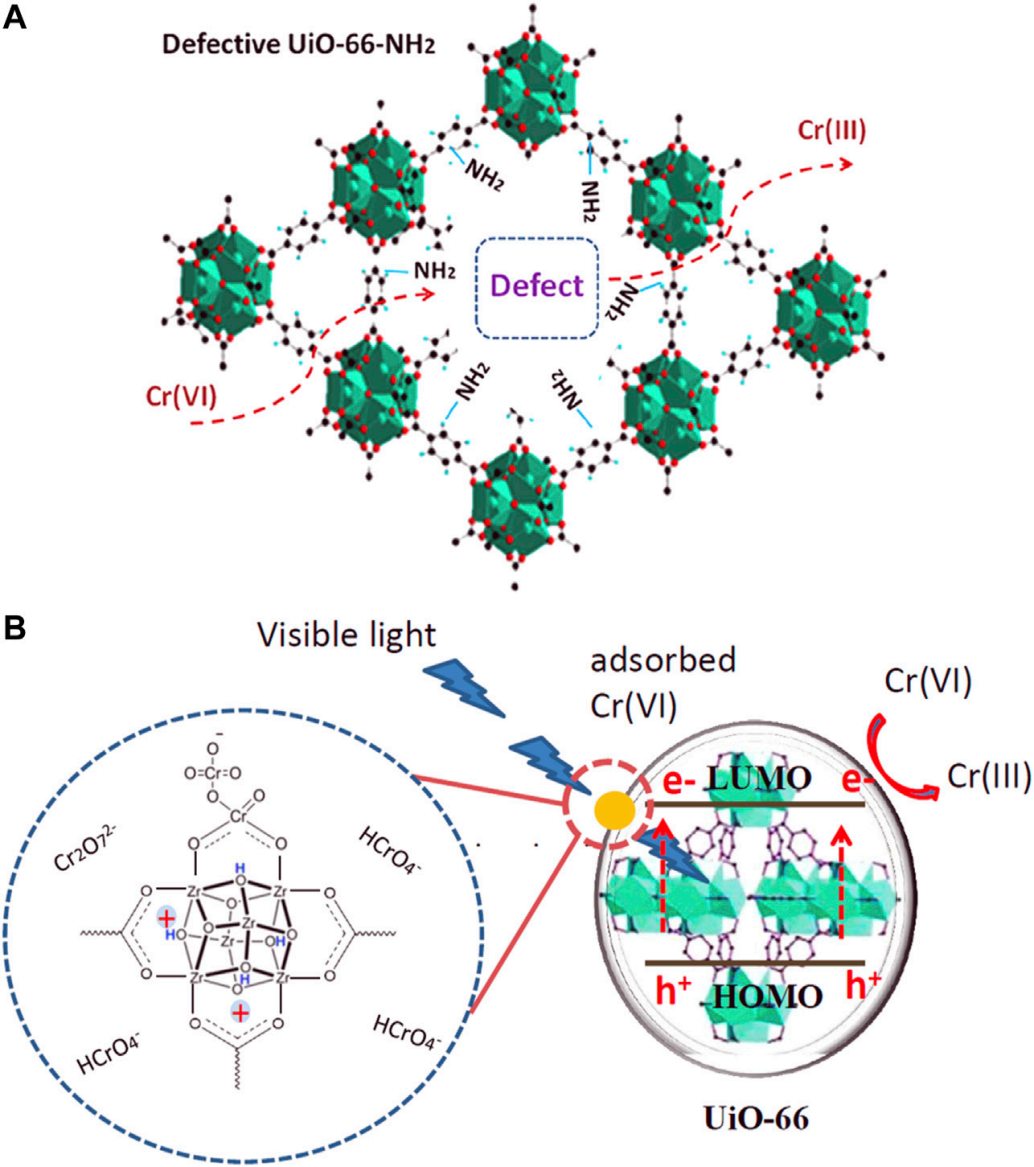
**(A)** UiO‒66‒NH_2_ (Ti) photocatalytic degradation Cr (VI) under visible light (Feng et al., 2019). **(B)** Mechanism of Cr (VI) reduction under visible light with defect UiO‒66 (Chen et al., 2019).

**FIGURE 11:**
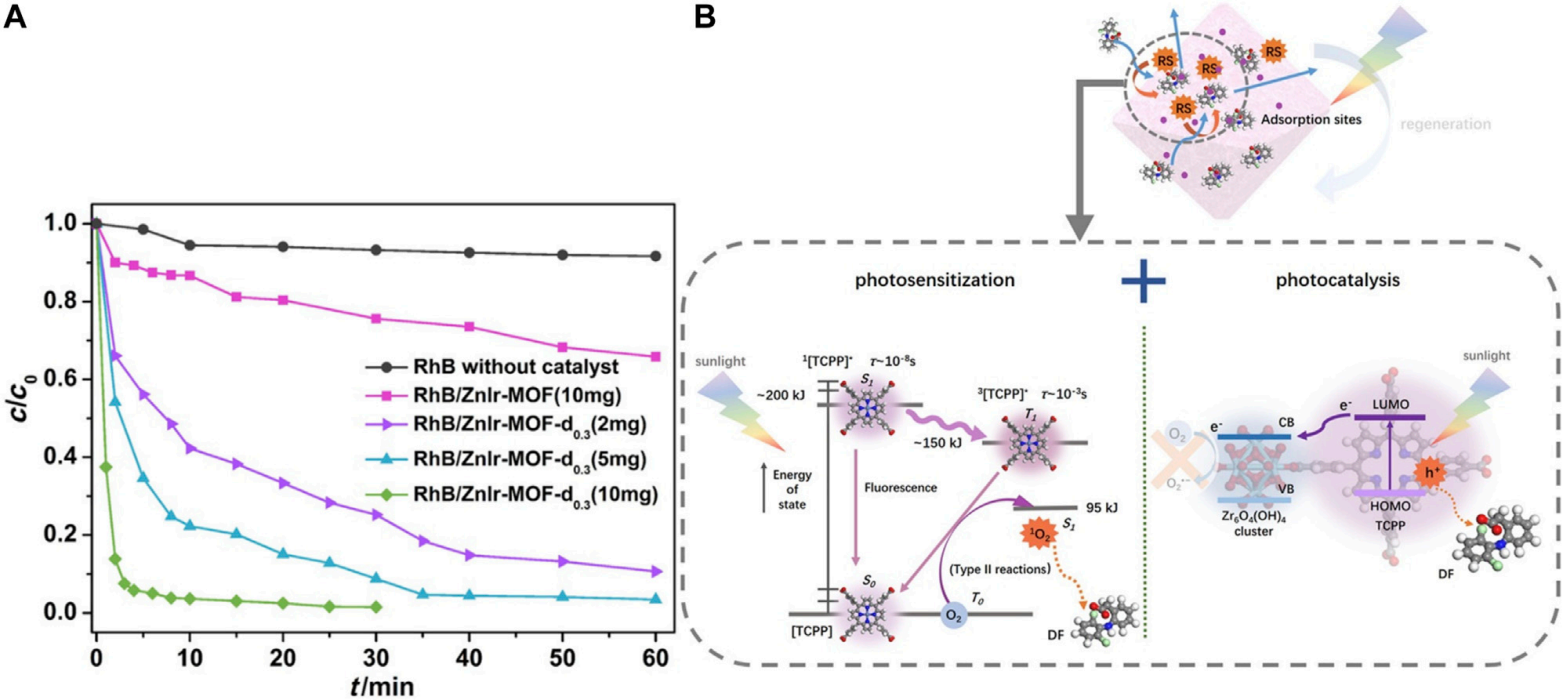
**(A)** ZnIr‒MOF photocatalytic degradation RhB (Fan et al., 2017) and **(B)** mechanism of DF degradation by TCPP@UiO‒66 (Gao et al., 2020).

(1) Metal ions Defective MOFs can facilitate toxic metal ions removal under visible light. Feng et al. chose the ZrOCl_2_·8H_2_O as a Zr precursor to synthesize UiO‒66‒NH_2_ combined with the post-synthetic treatment of Ti substitution (Feng et al., 2019). This highly defective framework and more ‒NH_2_ groups exposed on the surface improved the photocatalytic reduction of Cr (VI) under visible light. ‒NH_2_ groups exposed on the surface facilitated the light absorption ability while the defect structure decreased the recombination rate of photogenerated electron‒hole pairs, thus exhibiting a better photocatalytic performance. See Figure 10A. Adsorption is an important factor that affects the photocatalytic degradation rates of defective MOFs. Another similar work was reported by Chen et al. See Figure 10B. UiO‒66 was synthesized by a nonsolvent method with ZrOCl_2_·8H_2_O as a Zr precursor with abundant defects. Results show the best photocatalytic performance mainly due to the highest Cr (VI) adsorption capacity and the more positively charged framework, which facilitates the adsorption of Cr (VI) and desorption of cationic Cr (III) and the electron transfer from UiO‒66 to Cr (VI) ions (Chen et al., 2019).
(2) Organic pollutants As discussed in the above section, a series of defective iridium‒containing MOFs were designed by doping heterostructured linker Ir‒BH_3_. This defect structure not only improves the adsorption ability but also increases the photocatalytic efficiency of organic dye significantly, see Figure 11A. Fan et al. reported that the combination of Ir ligand and Zn_4_O cluster makes the band gap of MOF lower than that of many typical inorganic semiconductors (Fan et al., 2017). By studying the solid‒state fluorescence properties of MOF and ligand (318 nm excitation), they found that defective ZnIr‒MOF has excellent light collection ability in a wide visible light region. Rhodamine B can be degraded to 34% after 60 min by ZnIr‒MOF. However, RhB (1.56 × 1.23 × 0.42 nm) can be degraded completely (100%) within only 10 min by defective ones. This improvement can be attributed to the presence of mesopores, which can facilitate the mass transport process of organic compounds. See Figure 11A.
(3) Emerging Contaminants Figure 11B demonstrates how diclofenac (DF) can be effectively removed by TCPP@UiO‒66 which have been synthesized by large mixed‒linker approach. Gao et al. found that electrostatic interaction, Lewis acid− base interaction, π−π interaction, hydrogen bonding, and anion−π interaction were possible adsorption mechanisms. In the degradation process, the energy transfer from TCPP to triplet oxygen and the electron transfer from TCPP to Zr clusters contributed to the degradation of DF. Moreover, h^+^ is also a key reactive species (Gao et al., 2020). Table 2 lists the representative removal mechanisms of target compounds by defective materials.

**TABLE 2:** Mechanisms of target compounds removal by defective materials.

MOF	Removal	Pollutant	Mechanism	Ref.
UiO‒66	Adsorption	Uranium	—	Yuan et al. (2018)
UiO‒66	Adsorption	Arsenic	Coordination interaction	Assaad et al. (2020)
	Adsorption	Platinum	Electrostatic interaction	Lin et al. (2019)
			Ion exchange	
ZnIr‒MOF	Adsorption	N‒butanol	Size‒exclusion effect	Fan et al. (2017)
UiO‒66	Adsorption	Organophosphorous	—	Harvey et al. (2018)
UiO‒66	Adsorption	Coomassie bright blue	Size‒exclusion effect	Cai and Jiang (2017)
UiO‒66	Adsorption	Safranine T	Size‒exclusion effect	Wang et al. (2016)
UiO‒66	Adsorption	Rhodamine B	Size‒exclusion effect	Hu et al. (2019)
UiO‒66	Adsorption	Ibuprofen	Electrostatic interaction	Lin et al. (2018)
	Adsorption	Ketoprofen	Electrostatic interaction	Lin et al. (2018)
	Adsorption	Naproxen	Electrostatic interaction	Lin et al. (2018)
	Adsorption	Indomethacin	Electrostatic interaction	Lin et al. (2018)
	Adsorption	Furosemide	Electrostatic interaction	Lin et al. (2018)
	Adsorption	Salicylic acid	Electrostatic interaction	Lin et al. (2018)
UiO‒66	Adsorption	PFOS	Electrostatic interaction	Clark et al. (2019)
UiO‒66	Adsorption	Roxarsone	Coordination interaction	Li et al. (2016)
UiO‒66	Adsorption	Roxarsone	Coordination interaction	Xu et al. (2017)
			Hydrogen bonds	
	Adsorption	P‒arsanilic acid	Coordination interaction	Xu et al. (2017)
			Hydrogen bonds	
UiO‒66‒NH_2_	Photocatalysis	Chromium	—	Feng et al. (2019)
UiO‒66	Photocatalysis	Chromium	—	Chen et al. (2019)
ZnIr‒MOF	Photocatalysis	Rhodamine B	—	Fan et al. (2017)
TCPP‒UiO‒66	Photocatalysis	Diclofenac	—	Gao et al. (2020)

#### Electrocatalysis

**FIGURE 12:**
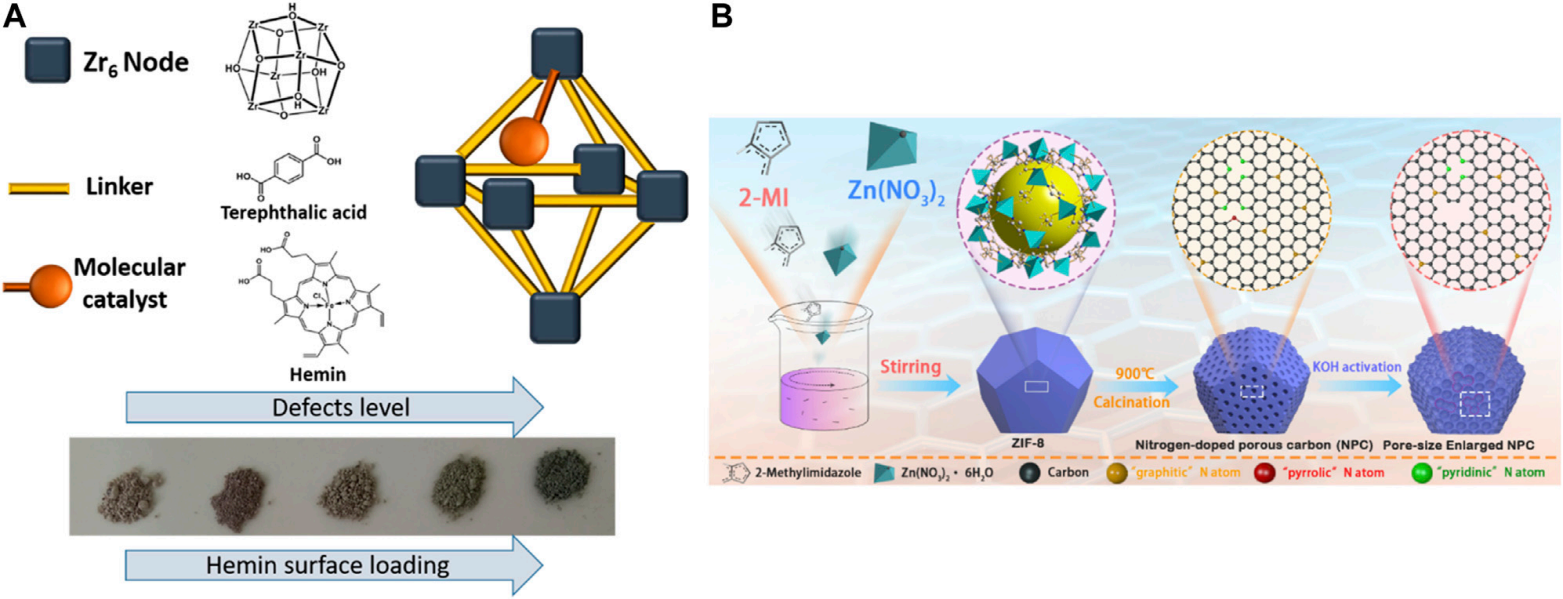
**(A)** Fe‒porphyrin‒based (Hemin)‒modified UiO‒66 (UiO‒66@Hemin) made by defective UiO‒66 (Shimoni et al., 2019). **(B)** Schematic diagram of the synthesis of the pore size‒enlarged NPC nanomaterials derived from ZIF‒8 precursors (Zhu et al., 2019).

Compared to other applications, there is a limited amount of research on the electrocatalysis degradation process by defective MOFs. There is an increasing need for introducing new approaches to tune and accelerate the rate of charge transport in MOFs. An ideal catalyst should have a large specific surface area with abundant active sites, high porosity, and high charge transfer ability. However, many chemically stable MOFs have limited charge transport abilities or poor conductivity. To solve this problem, several approaches have been reported: 1) MOF is used as a self‒sacrificing precursor or template to prepare porous carbon‒based composites with highly dispersed inorganic nanophases as catalytic active centers. 2) Charge transport through MOF‒installed conductive polymers. 3) Incorporated conductive guests. 4) Create two‒dimensional MOFs (Falcaro et al., 2014; Chakraborty et al., 2021). Although the work of MOF electrocatalytic degradation of pollutants has not been reported, this related publication will offer more guidance for future applications. Recently, there has been an increasing number of studies concerning the strategy to improve the electrocatalysis properties based on the defect structures. By adding HCl in the process of UiO‒66 synthesis, defective UiO‒66 can was synthesized successfully by Shimoni et al. These defective sites can serve as the postmodified ligand (Fe‒porphyrin (Hemin)‒based molecular catalyst) anchoring sites. Therefore, these defective sites incorporating conductive guests can increase the charge transfer rate by an order of magnitude. See Figure 12A (Shimoni et al., 2019).

Another strategy to improve the electrocatalysis properties is to synthesize MOF‒derived NPC nanomaterials. Zhu et al. developed a MOF (ZIF‒8)‒derived N‒doped porous carbon (NPC) nanomaterial as a highly active oxygen reduction reaction (ORR) catalyst. The NPC exhibits a comparable ORR activity, higher stability, and better tolerance to methanol compared with the commercial Pt/C. The density functional theory results show that N‒doped carbon along with the defects is more favorable for ORR compared with N‒doped carbon because the presence of defects leads to enhanced O adsorption ability and promotes the ORR process. See Figure 12B (Zhuet al., 2019).

## Future Perspectives

Hazardous pollutants (e.g., metal ions, pharmaceuticals and personal care products, PFOS, etc.) exist worldwide in aquatic environments because of edomestic or industrial discharge. They can pose considerable threat to human health and the ecosystem when they are released into the environment. For water treatment processes, there is an increasing demand for highly effective adsorption and degradation materials. Nanotechnology will open new ground and transform how water is purified.

Recently, the application of MOFs has increased in many fields. This coordination polymer will be very promising as an ideal adsorbent/catalysis because of the high porosity, large surface area, and tunable pore size. However, compared to the application in other fields, the studies for water treatment by MOFs still need more effort. Developing water stable and highly effective MOFs is crucial. In recent works, most of the efforts have been done on existing MOFs such as UiO‒66s and MILs. These MOFs show some excellent performance in several areas, including catalysis and separation. For a water treatment process, the selected materials need to be both relatively water stable and highly efficient. One of the strategies is to make the existing framework perform at its fullest capacity. There are major challenges in water treatment processes. For example, MOFs are inaccessible for some target guest compounds in water because of the mass transfer problem. One possible approach to achieve this purpose is defect engineering. The vacancies in defective MOFs could facilitate the purification process in the following aspects:

1) Mesoporous structure inside might enhance the mass transfer in adsorption and catalysis processes. 2) Make the materials more hydrophilic in water. 3) Offer CUSs that can facilitate the catalytic reaction selectively. 4) Introduce strong chemical interactions, such as electrostatic interaction. 5) Reduce the material price by alternating to cheaper linkers.

Therefore, if the defect structure can be created in a controllable manner and precisely characterized, defect engineering can be applied to improve current nanomaterials. Overall, defect engineering can excavate hidden active sites in MOFs. We believe this strategy can contribute to the development of future water treatment processes.
